# Machine learning based lineage tree reconstruction improved with knowledge of higher level relationships between cells and genomic barcodes

**DOI:** 10.1093/nargab/lqad077

**Published:** 2023-08-21

**Authors:** Alisa Prusokiene, Augustinas Prusokas, Renata Retkute

**Affiliations:** School of Natural and Environmental Sciences, Newcastle University, Newcastle upon Tyne NE1 7RU, UK; Independent researcher, London, SW7 2BX, UK; Department of Plant Sciences, University of Cambridge, Downing Street, Cambridge CB2 3EA, UK

## Abstract

Tracking cells as they divide and progress through differentiation is a fundamental step in understanding many biological processes, such as the development of organisms and progression of diseases. In this study, we investigate a machine learning approach to reconstruct lineage trees in experimental systems based on mutating synthetic genomic barcodes. We refine previously proposed methodology by embedding information of higher level relationships between cells and single-cell barcode values into a feature space. We test performance of the algorithm on shallow trees (up to 100 cells) and deep trees (up to 10 000 cells). Our proposed algorithm can improve tree reconstruction accuracy in comparison to reconstructions based on a maximum parsimony method, but this comes at a higher computational time requirement.

## INTRODUCTION

Single-cell lineage tracing, reconstructing the relationship between individual dividing cells in a tissue or organism, has the potential to improve our understanding of many biological processes, including the major transitions in evolution (1), development from the founding zygote to a complex organism (2), stem cell properties in tissue (3), cancer cell differentiation (4) or metastasis (5), and pathways of tumour evolution (6).

The first full development lineage tree was traced for the embryonic cells of the nematode *Caenorhabditis elegans* by sketching the events of cell division and development histories observed directly through light microscopy (7). Recently, platforms based on cell barcoding were proposed for tracking the lineage of individual cells at a high resolution. An integrase-based synthetic barcode system, intMEMOIR, uses the serine integrase Bxb1 to perform irreversible random modification of DNA recording arrays that can be read out using fluorescence *in situ* hybridization imaging (8). Fluorescent reporter assays allow the rapid characterization of these recording units (9). This experimental system is coupled with a time-lapse movie of the cells as they divide to provide a ground truth lineage tree (8). Another technique, substitution mutation-aided lineage tracing (SMALT) system, used a 3-kb readout sequence with 16 iSceI binding motifs to map single-cell resolution cell phylogenies during organ development (10). Additional strategies to achieve lineage tracing at single-cell resolution have been developed in the past few years: integration barcodes (designed as short DNA fragments placed in an expressed locus) and polylox barcodes (comprising a DNA cassette with multiple loxP sites in alternating orientations) (11). These, and future, technical advancements have to be matched by progress in relevant computational methods (12).

Various statistical methods for reconstructing gene trees and species trees (13,14) have been developed over the last few decades, but these methods have not been widely used on time and individual-cell resolved datasets. Mutations induced by cell barcoding methods are irreversible, which is different from the somatic mutations accumulated during mitotic cell division (8). Previously, we have developed a machine learning (ML) approach for cell lineage reconstruction, with results submitted to the Allen Institute Cell Lineage Reconstruction DREAM Challenge (15). This was the best-performing algorithm for the reconstruction of *in vitro* cell lineages of trees with <100 cells. It reached higher accuracy than other methods, such as distance-based method DCLEAR (16), maximum parsimony based method Cassiopeia-integer linear programming (17) and Cassiopeia-Greedy (17). Our proposed framework was based on embedding single-cell data into a feature space in order to train an ML algorithm to predict the probability that cells are siblings.

In this study, we expanded this method by training an ML model to predict higher level relationships between cells. We refined our methodology to be applied to any number of possible edit states. To evaluate the performance of the algorithm, we used two sets of *in silico* data: (i) shallow trees (up to 100 cells), simulated using parametrization based on *in vitro* dataset for mouse embryonic stem cells (15); and (ii) deep trees (up to 10 000 cells), simulated using parametrization based on *in vitro* dataset for fly organ development (10). Further, we reconstructed trees of *in vitro* dataset for mouse embryonic stem cells (15). We measured the accuracy of lineage reconstruction using the normalized Robinson–Foulds (RF) score. For trees with up to 100 cells we additionally calculating four metrics: normalised Robinson Foulds score (RF), triplet score (TRP), quartet score (QRT), and clustering information score (CLI). We compared our reconstruction with a maximum parsimony method. We found that our proposed algorithm has an advantage over the maximum parsimony method in terms of reconstruction quality for both shallow trees and deep trees.

## MATERIALS AND METHODS

### The *in vitro* datasets

#### Mouse embryonic stem cell colonies

The data were obtained as part of the Allen Institute Cell Lineage Reconstruction Challenge through Synapse ID syn20692755 (15). In the experiment, the recording array (barcode) consisted of *L* = 10 recording units (8). Each recording unit was in one of three states: ground state (represented as ‘1’), a deletion (represented as ‘0’) or an inversion (represented as ‘2’) of the DNA sequence. Each colony was started from an individual cell and colony growth was observed for 48 h. The experimental data comprise (i) an array of intMEMOIR readouts as a text file (also called matrix) and (ii) the ground truth lineage for the colony. Tree-like data structures were provided as a Newick file (18). The ground truth cell lineage trees were obtained from video-microscopy data (8).

#### Fly organ development

A SMALT system used a barcode with 16 iSceI binding motifs present with equal distance throughout the sequence (10). The recording array consisted of *L* = 2943 recording units. Each recording unit was in one of two states: ground state (represented as ‘0’) or mutated state (represented as ‘1’). Binary sequence data were available for two specimens: one had 5002 cells and other 5420 cells. The datasets correspond to fly organ development from embryo to late-third instar larvae (10).

### The *in silico* datasets

#### Stochastic simulation of cell division and barcode editing

**Figure 1.**
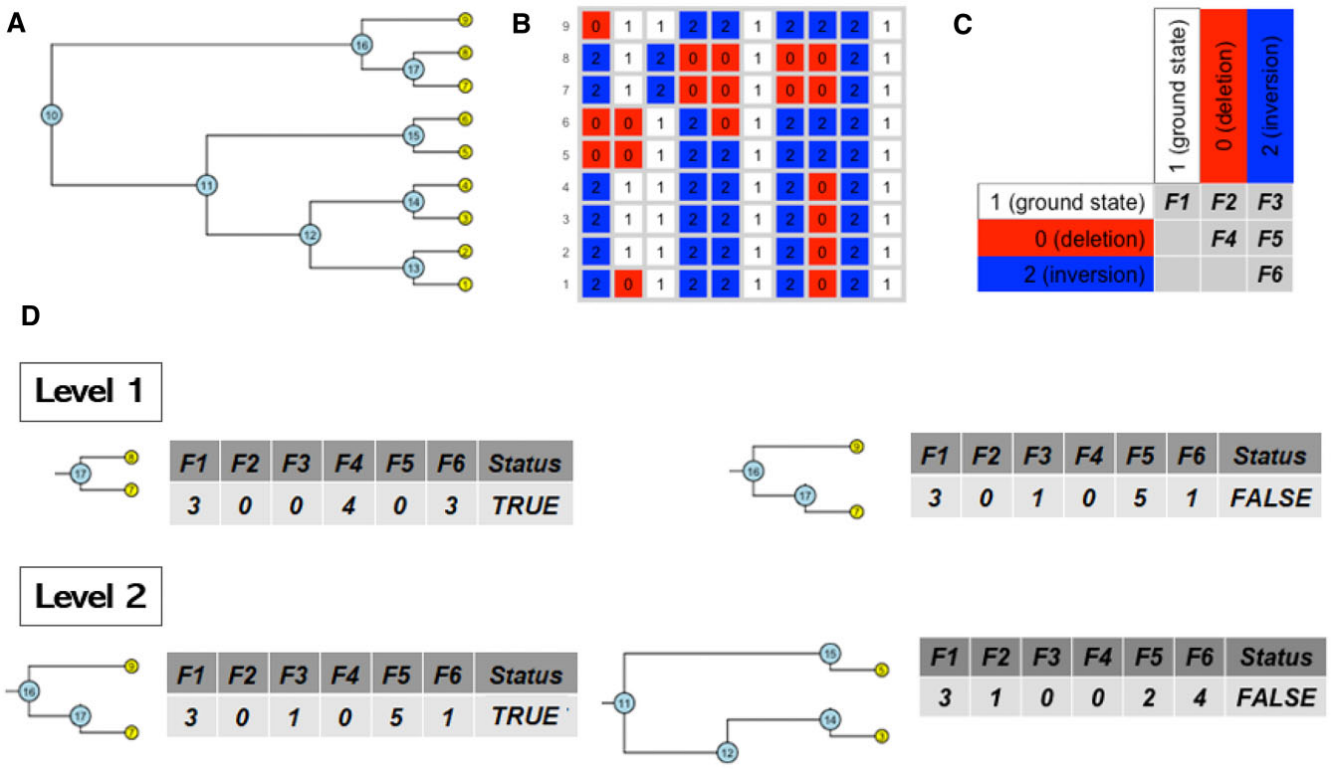
The ML approach for lineage tree reconstruction. (**A**) Ground truth lineage. Here, we have a colony with nine cells; each cell corresponds to a tip of the lineage tree (yellow circles). All cell pairs have a common node (blue circles), showing their level of relatedness. (**B**) Corresponding barcode states: rows correspond to cells and columns to recorder units. A unit can be at the ground state (encoded as ‘1’), deletion (encoded as ‘0’) or inversion (encoded as ‘2’). (**C**) Feature construction is based on pairwise comparison of barcodes: number of units that have not been edited (F1); number of units that have the same edit (F4 and F6); number of units that have single edit (F2 and F3); and number of units that have different edits (F5). (**D**) Two examples of embedding single-cell data into a feature space for ML training. The upper row corresponds to level 1 (sibling cells) and the lower row corresponds to level 2 (cousin cells). State indicates whether relationship is true or false.

Accumulation of stochastic mutations during cell division was simulated similarly to (8,19). Each cell colony starts from an individual cell carrying an unedited array with length of *L* units. The model assumes that cells divide synchronously and at a constant rate. The initial cell then undergoes a series of cell divisions (Figure 1A). After *d* divisions, the colony consists of *N* cells, where *N* = 2^*d*^.

After every cell division, each unedited target can mutate with a given probability *μ* to one of several possible edited states *s* ∈ *S*. The state is chosen according to a probability α_*s*_. The process of recording is irreversible, and once a recording unit is edited, it can no longer change (Figure 1B).

The probability that a recording unit has not been edited after *d* divisions is equal to $P_{{\rm not}\_{\rm edited}}(d, \mu , \boldsymbol{\alpha })=\left(1-\mu \right)^{d}$. The ground truth lineage could provide necessary information on what is an expected lower number of divisions each cell has undergone in a colony, for example by counting the number of ancestors for phylogenetic nodes.

The probability of an edited unit going to state *s* is denoted by *α*_*s*_, such that $\sum\nolimits_{s=1}^{|S|}=1$, where *S* is a set of all possible edits and |*S*| denotes the size of set *S*. In the case of the mouse embryonic stem cell colonies dataset, |*S*| = 2 and *S* = {0, 2}, and in the case of the fly organ development dataset, |*S*| = 1 and *S* = {1}. The probability that a recording unit is in state *s* at division *d*, given that it was in the unedited state at division *d* − 1, is equal to $P_{{\rm edited}}(d, \mu , \boldsymbol{\alpha })=\left(1-\mu \right)^{d-1} \mu \alpha _s$. As the state values of lineage tree internal nodes are not available, we do not know which division incurred the editing. Therefore, the probability of observing the recording unit in state *s* after *d* generation becomes $P_{{\rm edited}} (d, \mu , \boldsymbol{\alpha })=\sum\nolimits_{k=1}^{d} \left(1-\mu \right)^{k-1} \mu \alpha _s$.

The total likelihood is equal to


(1) $$\begin{eqnarray*} L (\mu , \boldsymbol{\alpha }) = \prod _{c=1}^C \prod _{i=1}^{n_c} \prod _{l=1}^{L} P_{o_{c,i,l}}(d_{c,i}, \mu , \boldsymbol{\alpha }), \end{eqnarray*}$$

where *C* is the total number of colonies, *n*_*c*_ is the number of cells in a colony *c*, *d*_*c*,*i*_ is the number of divisions for cell *i* in colony *c*, *o*_*c*,*i*,*l*_ is the observed state for unit *l* in cell *i* and colony *c*, and $o_{c,i,l}=\lbrace {\rm not}\_{\rm edited}, {\rm edited}\rbrace$.

For parameter estimation, we used the R package AMISEpi, which has an implementation of adaptive multiple importance sampling for Bayesian analysis (20). We set the priors to be uniformly distributed: *μ* ∼ *U*[0, 1] and *α*_*i*_ ∼ *U*[0, 1].

#### Simulation of shallow trees (up to 100 cells)

We used parameters (mutation rate and probability of mutations) estimated from the *in vitro* mouse embryonic stem cell dataset (15). We used data on all colonies for parameter estimation.

#### Simulation of deep trees (up to 10 000 cells)

We used parameters (mutation rate) estimated from the *in vitro* fly organ development dataset (10). We have used both datasets to estimate the mutation rate per division/target. We assumed that there were 13 divisions, which was closest to the number of cells available for dataset (2^13^ = 8192).

### Lineage tree reconstruction using the ML approach (AMbeRland-TR)

#### Feature engineering

For illustration purpose, we assume that the barcode units can be three possible states: ground state, a deletion or an inversion (Figure 1B). We are proposing the following classes of features based on pairwise comparison of barcodes: number of units that have not been edited (F1); number of units that have the same edit (F4 and F6); number of units that have single edit (F2 and F3); and number of units that have different edits (F5) (Figure 1C).

This approach can be extended to any number of possible edited states by including all possible pairwise combinations of the extended record set $\lbrace {\rm not}\_{\rm mutated}, S\rbrace$ as predictors for the ML model. This should produce (|*S*| + 1)! predictors.

We have used the R package phangorn (21) to extract information on relatedness between cells from the ground truth cell lineage trees. For each level of the tree, this produced two lists of cell pairs for each colony: cells that share an ancestor at level *t* and cells that do not share an ancestor at level *t*. Two examples of embedding single-cell data into a feature space for ML training are shown in Figure 1D. The upper row corresponds to level 1 (sibling cells) and the lower row corresponds to level 2 (cousin cells). State indicates whether relationship is true or false.

#### ML training, prediction and interpretation

We used a gradient boosting machine (GBM) to implement the outlined ML approach. All calculations were performed in R using package *gbm* (22). The following options were used to train the GBM model: distribution = ‘bernoulli’; n.trees = 1000; interaction.depth = 10; n.minobsinnode = 5; cv.folds = 5; and train.fraction = 0.5.

We use the relative importance of features, partial dependence plots and individual conditional expectation plots for model interpretation. Relative importance is based on the number of times a predictor is selected when training the model (23). Higher values of relative importance indicate larger influence on the response. Individual conditional expectation curves visualize the partial relationship between the predicted response and a feature for individual datasets (24). Partial dependence plots show the relationship averaged over all observations, which makes it easier to extract expected trends (24).

#### Clustering

We applied a custom hierarchical clustering method for building a cell lineage tree from predicted probabilities. Clustering begins at the lowest tree level, where all clusters contain an individual cell. Each possible cell pair is then ranked according to the predicted probability that they share an ancestor at this level. At consecutively increasing levels, pairwise comparison is performed between each lower level cluster, where the calculated probability is the maximum between any elements of the two clusters. Cluster pairs are ordered again according to this probability and are assumed to have the same parent node if its value is above the estimated threshold for this level. This process is repeated until one or two clusters are left. We assume only binary trees.

### Lineage tree reconstruction using a maximum parsimony tree reconstruction method

Maximum parsimony based methods try to find the minimum number of changes necessary to describe the data for a given tree. We performed a maximum parsimony reconstruction using the R package phangorn (21). Initial tree was required to start the maximum parsimony tree search. This was done using the Hamming distance between barcodes [package DescTools (25)] and unweighted pair group method with arithmetic mean clustering (26). We set the method as ‘fitch’ and minimum number of iterations in the ratchet as 100.

### Scores

We assessed the accuracy of lineage tree reconstruction using four metrics: normalized RF score, TRP score, QRT score and CLI score. All scores have values between 0 and 1, with smaller values indicating larger similarity between ground truth lineage tree and reconstructed lineage tree.

#### Normalized RF score

The RF distance counts the number of splits that are unique to one of the two trees (27). An RF distance of 0 indicates that all splits in both trees are the same. We used the *RF.dist* function from the R package phangorn to compute the normalized RF score (21).

#### TRP score

The TRP distance counts the number of subtrees of three taxa that are different in the two trees (28). The TRP distance was calculated using the tqDist algorithm (29) implemented in the R package Quartet (30). The TRP score was calculated by dividing the TRP distance by the total number of triplets shared between the two trees, i.e. $\binom{N}{3}$ (29).

#### QRT score

The QRT distance enumerates all subsets of leaves of size 4 and counts how often the topologies induced by the four leaves agree in the two trees (31). QRT divergence was calculated using the tqDist algorithm (32,33) implemented in the R package Quartet (30). The QRT score was calculated as one minus QRT divergence.

#### CLI score

CLI distance is a generalized RF metric based on the information content of the largest split (34). To compute the CLI score, we use the function *ClusteringInfoDistance* with the option *normalize = TRUE* from the R package TreeDist (35).

### Set-up for lineage tree reconstruction

#### In silico shallow trees

To determine the effect of varying the number of possible states and the number of recording units on the accuracy of lineage reconstruction, we performed simulations with the number of states varied between 2 and 6 and a recorder carrying either 10 or 20 units. For each configuration, we simulated 1000 lineage trees that were used for ML training, and an additional 100 lineage trees that were used for the testing of lineage reconstruction methods. For each simulation, we sampled the depth of lineage from the empirical distribution associated with the mouse embryonic stem cell dataset and editing rate from the fitted posterior distribution.

#### Mouse embryonic stem cell colonies

We used the same partition of the data as in (15), i.e. array readout data from 76 colonies along with the corresponding ground truth lineages as the training set and array readout data from 30 cell colonies as a testing set for accuracy evaluation.

#### In silico deep trees

We simulated division and target editing for 10 and 12 divisions, which produced colonies with 1024 and 4096 cells, respectively. For trees with 1024 cells, we trained an ML model using data obtained from 10 individual trees, but a single simulated tree was used to train the ML model for the case with 4096 cells.

## RESULTS

### Comparison of tree reconstruction accuracy scores

**Figure 2.**
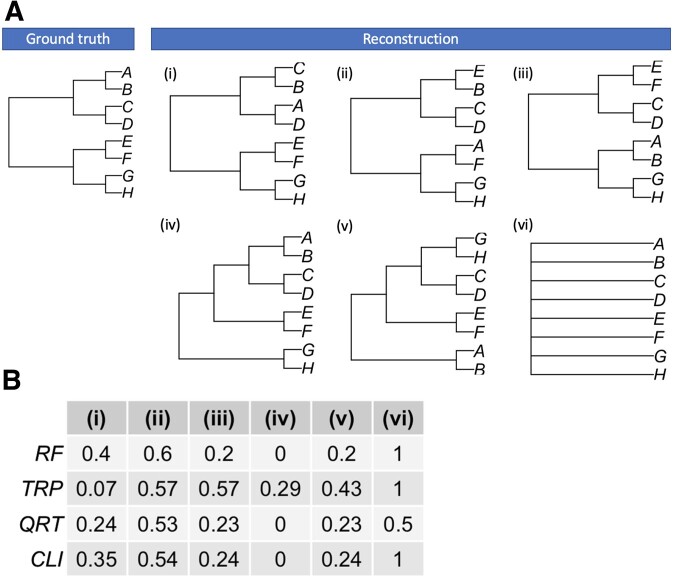
Comparison between scores for a colony with eight cells. (**A**) Ground truth lineage tree and six possible reconstructed tree topologies (i–vi). (**B**) Corresponding scores for each reconstruction from panel (A): normalized RF score, TRP score, QRT score and CLI score.

First, we investigated performance of scores under conditions where a lineage tree is reconstructed erroneously. Figure 2 shows an example how scores compare for a tree with eight cells and few possible reconstructions. In case (i), cells *A* and *C* (which are cousins) have been assigned incorrectly. This resulted in the normalized RF score equal to 0.4, but had a small effect on the TRP score (0.07). In case (ii), we assign cells *A* and *E* incorrectly, which increases all scores, including the TRP score, to have values between 0.53 and 0.6. If two pairs of cells were assigned incorrectly [case (iii)], the normalized RF score is reduced by a factor of 3 and the QRT score by a factor of 2, but the value of the TRP score stayed the same as in case (ii). Next, we assumed that the number of divisions was estimated incorrectly for a pair of cells (*G*, *H*) [case (iv)]. Although this results in a different structure of the tree, only the TRP score detected the discrepancy, giving a value of 0.29, with all other metrics at value 0. So, if the quality of reconstruction would be judged solely by the normalized RF score, QRT score or CLI score, this would erroneously suggest that case (iv) is a perfectly reconstructed tree. If further two pairs of sibling cells are swapped [case (v)], this increases scores by 0.2–0.23 points. Finally, if all cells are reconstructed as sibling cells [case (vi)], the QRT score would be equal to 0.5, indicating that half of all possible quartets were reconstructed correctly.

### Estimating mutation rates

#### The mouse embryonic stem cell dataset

**Figure 3.**
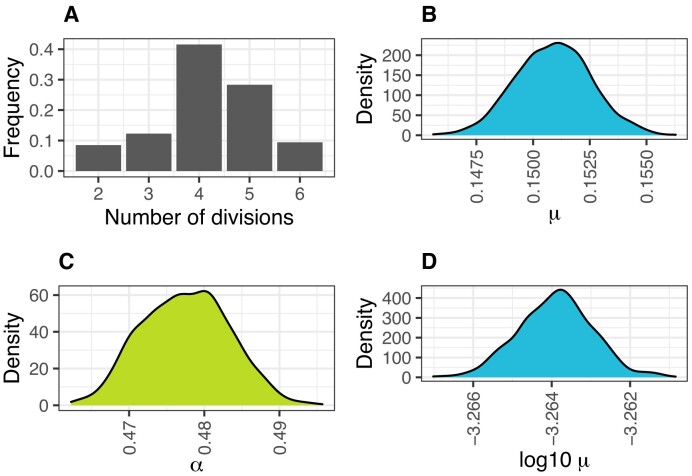
Parameter estimation. (**A**) Distribution of the number of divisions in the mouse embryonic stem cell experiment dataset. (**B**) Posterior distribution of editing rate (*μ*) in the mouse embryonic stem cell experiment dataset. (**C**) Posterior distribution of the probability that an edited unit has the state ‘2’ (*α*) in the mouse embryonic stem cell experiment dataset. (**D**) Posterior distribution of editing rate (*μ*) in the fly organ development dataset.

The resulting colonies had from 4 to 39 cells; in total, there were 1453 individual cells in the dataset. The distribution of the number of divisions in the ground truth lineage trees is shown in Figure 3A.

Posterior distributions for fitted editing rate and the probability that an edited unit has a state ‘2’ are shown in Figure 3B and C, respectively. We estimated the mean of marginal posterior distributions to be *μ* = 0.15 and *α* = 0.48. For simulation of cell division and record editing, we assume that all states have the same probability, i.e. *α*_*s*_ = 1/|*S*|.

#### The fly organ development dataset

Figure 3D shows the posterior distribution of mutation rate. We estimated the mean of marginal posterior distributions to be 0.0005. This is in agreement with (10), where it was concluded that ∼0.8–1.3 mutations were recorded on the readout sequence per cell generation.

### Lineage reconstruction of *in silico* dataset: shallow trees

**Figure 4.**
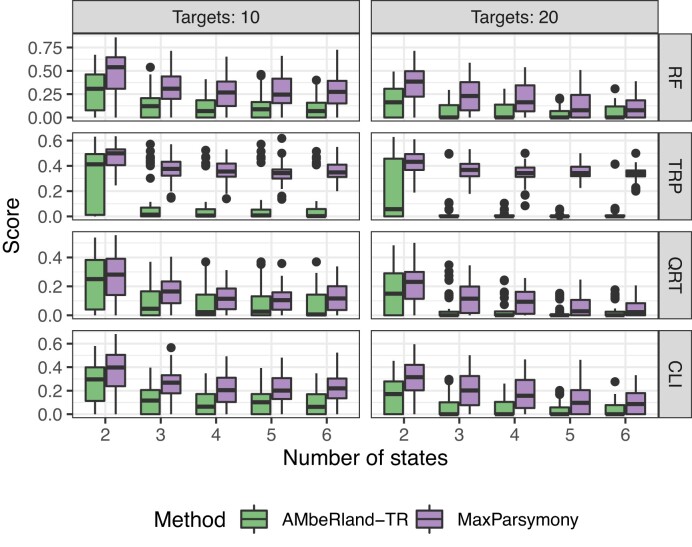
Lineage reconstruction accuracy for the *in silico* dataset, with *L* = 10 or *L* = 20 recording units, and the number of possible edits ranging from 2 to 6. Each bar summarizes the score of 100 lineage tree reconstruction tests. We denote our ML-based approach as ’AMbeRland-TR’ and lineage reconstruction using the maximum parsimony approach as ’MaxParsymony’. Accuracy metrics are normalized RF score, TRP score, QRT score and CLI score.

Results of simulations are shown in Figure 4. Our ML-based algorithm outperformed the maximum parsimony based method in all four metrics. Comparing mean performance, we found a 43–50% improvement in the normalized RF score, a 19–32% improvement in the QRT score and a 36–45% improvement in the CLI score.

We found that increasing the number of target units had a stronger effect on lineage reconstruction accuracy than increasing the number of states. For a recording array using 20 targets instead of 10 targets, there was a 62% improvement in performance for the QRT score, a 56% improvement in performance for both the normalized RF score and CLI score, and a 28% improvement in performance for the TRP score. When the number of editing states was increased from 2 to 5, there was a 17% improvement in the normalized RF score for a recording array using 10 targets. There was a 68% improvement in the normalized RF score for a recording array using 20 targets. There was a 30% improvement in the QRT score for a recording array using 10 targets and a 49% improvement in the QRT score for a recording array using 20 targets.

### Lineage tree reconstruction of the mouse embryonic stem cell dataset

**Figure 5.**
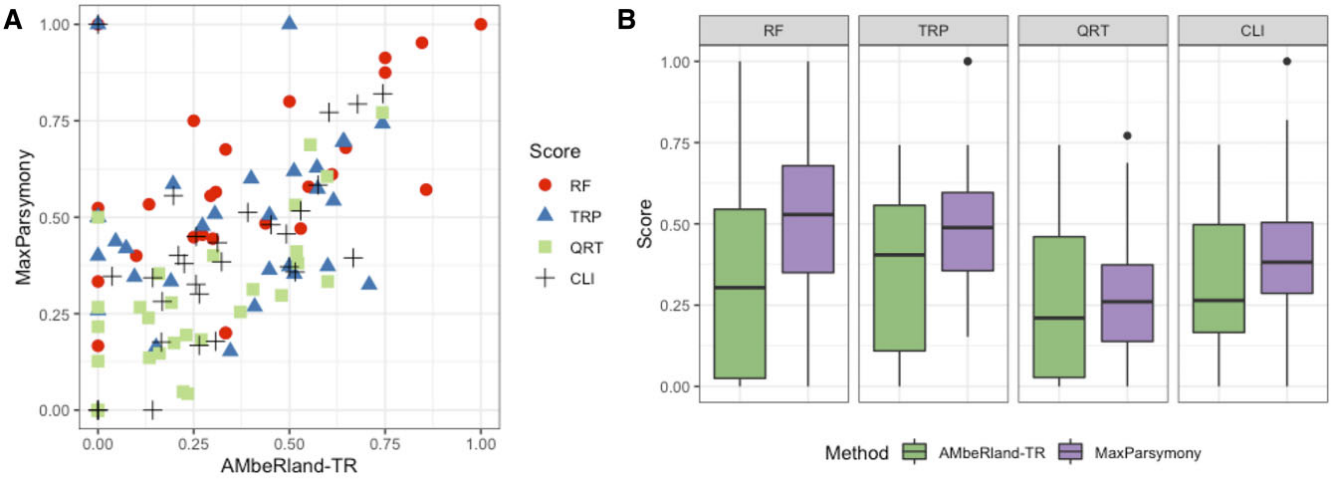
Lineage tree reconstruction accuracy for the mouse embryonic stem cell dataset: (**A**) pairwise comparisons and (**B**) distribution of scores. We denote our ML-based approach as ’AMbeRland-TR’ and lineage reconstruction using the maximum parsimony approach as ’MaxParsymony’. Accuracy metrics are normalized RF score, TRP score, QRT score and CLI score.

A reconstruction was computed from the test dataset consisting of 30 cell colonies using only the intMEMOIR array readout (8). Figure 5A shows a pairwise comparison between the two methods. Most of the points are above the diagonal, indicating that our ML-based algorithm outperformed the maximum parsimony based method for all four metrics. Comparing mean performance, we found a 17–23% improvement in the normalized RF score, a 7–37% improvement in the TRP score, a 7–11% improvement in the QRT score and a 12–15% improvement in the CLI score (Figure 5B).

**Figure 6.**
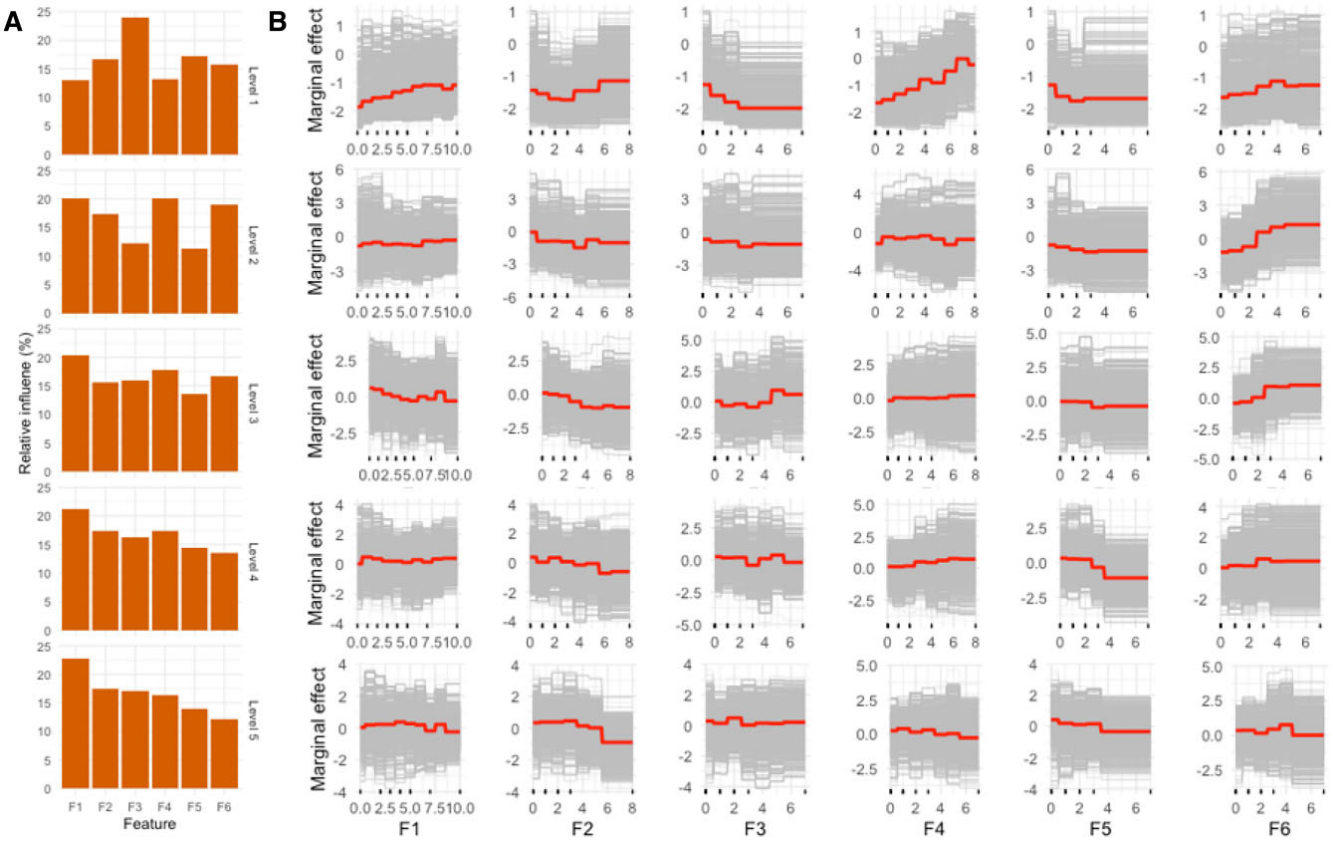
Interpretation of the model for the mouse embryonic stem cell dataset. (**A**) Relative importance of features. (**B**) Average partial dependence (red line) and individual conditional expectation (grey line) of cell relatedness probability on features. Rows correspond to levels of lineage tree hierarchy, with the lowest level (siblings) on the top.

Partial dependence plots and individual conditional expectation plots can be used to analyse the relationship between features and the response. It can be seen from partial dependence plots (red line) in Figure 6 (upper row) that the probability of cells being siblings decreases when the value of feature ‘F3’ increases up to 3. This has a biological meaning: the number of pairwise barcodes where one cell stayed in the ground state and the other had undergone editing cannot be high if the cells are siblings, and the highest probability is when there are no such barcodes. The same relationship is true for the feature ‘F5’: the probability of cells being siblings decreases with increasing numbers of pairwise barcodes where one cell had undergone deletion and the other inversion. There is a linear relationship between the probability of cells being siblings and the number of pairwise barcodes where both cells have undergone deletions (feature ‘F4’). These dependences for ‘F3’ and ‘F4’ become weaker when cells get further apart on the lineage tree (lower rows). The conditional expectation plots (grey lines) demonstrate that at the individual level, there are more complex relationships between features.

### Lineage reconstruction of *in silico* dataset: deep trees

**Figure 7.**
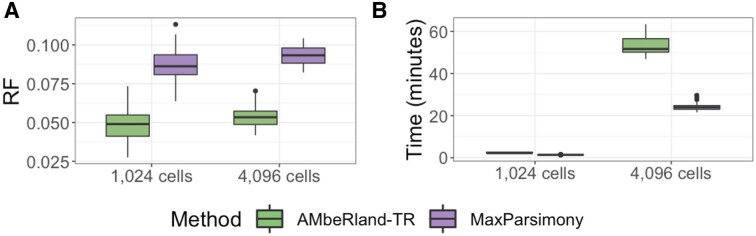
Lineage reconstruction for deep trees. (**A**) Normalized RF score. (**B**) Time requirements for the algorithm.

The performance of the AMbeRland-TR algorithm was consistent between reconstruction of trees with 1024 cells and 4096 cells, and in both cases outperformed the reconstruction by the maximum parsimony method (Figure 7A). On average, there was a 50% improvement in performance for the normalized RF score in comparison to the maximum parsimony method. Improvement in reconstruction quality comes at higher computational time requirements. It takes 54 min on average to reconstruct a tree with 4096 tips using the AMbeRland-TR algorithm, which is two times longer than the time required for the maximum parsimony method (Figure 7B).

### Computational requirements

**Figure 8.**
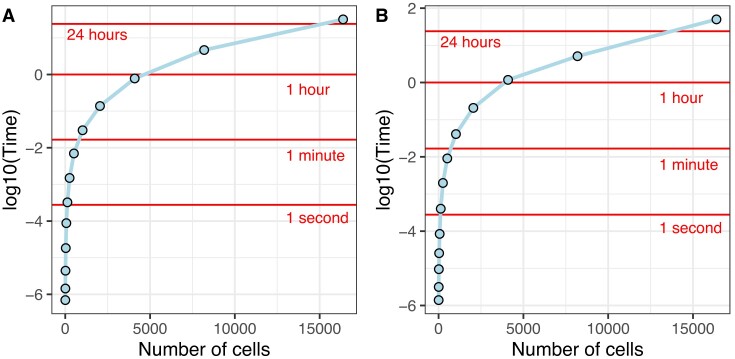
Time requirements for the algorithm. (**A**) Training data preparation and ML model training. (**B**) Testing data preparation and tree reconstruction.

The computational cost of the AMbeRland-TR algorithm has the following components: extracting features from barcode data; extracting relationships between cells from the ground truth trees; training the ML model for each tree level; and reconstructing trees for testing data. We have combined all procedure into two tasks: (i) training data preparation and ML model training; and (ii) testing data preparation and tree reconstruction. For the purpose of evaluating time requirements, we assume that training and testing data have only a single cell colony. For deep trees, as shown in the previous section, it is enough to train an ML model on single tree. For shallow trees, the training dataset should contain enough trees to accommodate variate of barcode combination, but this should not be a burden as training data preparation for shallow trees is computationally fast. Overall, time required for training and testing tasks was similar for a range of number of cells we investigate (Figure 8). For example, for a tree with 10 000 cells, it would take ∼24 h to train the models and 24 h for reconstructing a tree. All calculations were performed on a MacBook Pro with 2.4-GHz 8-core processor.

## DISCUSSION

Machine learning is becoming an important tool that has considerable potential in biology (36), genetics and genomics, including the annotation of sequence elements and epigenetic, proteomic and metabolomic data (37), or solving problems arising in population and evolutionary genetics (38). So far, it has been underutilized for lineage tree reconstruction. In this study, we introduced the framework for constructing features and training an ML algorithm for experimental systems based on mutating synthetic genomic barcodes. We explored how the barcoding configuration influenced the performance of the algorithm by using a collection of simulated and biological datasets.

There is no universal method to quantify topological similarities between lineage trees (39). The most widely used score is the RF score, but it is very insensitive to discrepancies at higher levels of lineage tree structures [Figure 2 (iv)]. Assigning the correct pathway cells undergo during organism or cancer development is necessary in order to understand tissue-type differentiation. The QRT score systematically showed lower values indicating higher similarity between ground truth and reconstruction, even when reconstructed lineage trees lacked any structure [Figure 2 (vi)]. Therefore, calculating a combination of accuracy scores is necessary in assessing the quality of lineage tree reconstruction.

Our *in silico* lineage reconstruction experiments showed that the ML-based approach is able to take advantage of high complexity of relationships between processes governing cell division and barcode editing. Under ideal conditions, i.e. no noise in observations and a large training dataset, they have outperformed other statistical approaches by 19–62% for scores evaluating various aspects of lineage reconstruction (Figure 4). It has also showed that when engineering the barcode system, it is more desirable to increase the number of recording units on barcode arrays than to increase the number of possible mutation states. However, increasing the number of edits from two states (i.e. single possible mutation) to three states (two possible mutations) could improve lineage reconstruction accuracy by at least 50% on average.

The two *in vitro* datasets represent different lineage tracing configurations: number of states (3 versus 2) and number of recording units (10 versus 2943). The ML approach performed better than the Hamming distance approach on both datasets. The difference was not as striking as for the simulated data. Possible explanations include the following: much smaller training dataset, larger heterogeneity between colonies or the presence of noise from experimental readouts.

From the model fitted to the mouse embryonic stem cell dataset, we can get insight into the functional relationship between experimentally observed barcode values and cell relatedness. For all levels of lineage tree hierarchy, we found that the features had a relative influence in the range from 11% to 24%; i.e. none of the six predictors had zero influence (Figure 6A). However, the ranking of features varied with the relationship level. When predicting whether cells are siblings (level 1), the highest relative influence was the number of pairwise barcodes where one cell was in the ground state and the other was inverted. This feature had lower influence for higher relatedness levels.

Our lineage reconstruction of the intMEMOIR dataset submitted to the Allen Institute Cell Lineage Reconstruction DREAM Challenge was the best-performing algorithm (mean RF score of 0.53 and TRP score of 0.52) (15). It was the best ranking method when benchmarked against a Bayesian phylogenetic framework (40). By training the ML model to predict higher level relationships between cells, we have been able to further improve performance. We achieved a mean RF score of 0.31 and a mean TRP score of 0.41 (Figure 5).

The area of lineage tracing is expanding fast, with new tools being developed at the experimental and computational levels. Jointly profiling DNA methylation, chromatin accessibility, gene expression and lineage information in single cells was made possible by developing an inducible lineage tracing mouse model with extremely large lineage barcode diversity (41). A computational pipeline allowing to predict cell lineages over several cell divisions solely from transcriptomic data alone was devised by leveraging genes displaying conserved expression levels over cell divisions (42). Another important direction is to understand cell fate transitions during development. An internal cellular clock could be recovered by integrating single-cell transcriptomics with lineage tracing (43,44). The ML approach has the potential to integrate barcode recordings with additional information, such as population dynamic parameters (40), single-cell gene expression (45), proteomics (46), microsatellite mutations (47) or clonal correlations (48).

A key limitation of our proposed approach is that it requires the ground truth dataset, i.e. recorded barcodes and tracked lineage histories. Except in the mouse embryonic stem cell experiment (15), such data are not available. One solution would be to train ML on simulated data. Having a global database with data on different lineage tracing configurations and results could be a starting point for accumulating knowledge as required for simulations. Under time and budget restrictions, having ground truth data on a single lineage tree makes it possible to train ML models, as our analysis on deep trees indicates. Future work should explore these possibilities and evaluate how to practically go about the process of training and improving ML models for reconstructing whole organ or even whole body lineage trees.

## Data Availability

Analysis code used in this study can be accessed at the following URL: https://github.com/rretkute/AMbeRlandTR (permanent DOI: 10.5281/zenodo.8227619).
